# Mapping suitable great ape habitat in and around the Lobéké National Park, South‐East Cameroon

**DOI:** 10.1002/ece3.7027

**Published:** 2020-12-05

**Authors:** Yisa Ginath Yuh, Paul K. N'Goran, Zacharie N. Dongmo, Wiktor Tracz, Elvis Tangwa, Michael Agunbiade, Hjalmar S. Kühl, Tenekwetche Sop, Chefor Fotang

**Affiliations:** ^1^ Warsaw University of Life Sciences (WULS‐SGGW) Warszawa Poland; ^2^ Eberswalde University for Sustainable Development (HNEE) Eberswalde Germany; ^3^ University of Concordia Montréal QC Canada; ^4^ Regional Office for Africa – Yaoundé Hub World Wide Fund for Nature (WWF) Yaoundé Cameroon; ^5^ Cameroon Country Program Office World Wide Fund for Nature (WWF) Yaoundé Cameroon; ^6^ Max Planck Institute for Evolutionary Anthropology (MPI EVAN) Leipzig Germany; ^7^ German Centre for Integrative Biodiversity Research (iDiv) Halle‐Leipzig‐Jena Leipzig Germany; ^8^ Department of Ecology Brandenburg University of Technology Cottbus Germany

**Keywords:** chimpanzees, forest management units, gorillas, habitat suitability, Lobéké National Park, MaxEnt

## Abstract

As a result of extensive data collection efforts over the last 20–30 years, there is quite a good understanding of the large‐scale geographic distribution and range limits of African great apes. However, as human activities increasingly fragment great ape spatial distribution, a better understanding of what constitutes suitable great ape habitat is needed to inform conservation and resource extraction management. Chimpanzees (*Pan troglodytes troglodytes*) and gorillas (*Gorilla gorilla gorilla*) inhabit the Lobéké National Park and its surrounding forest management units (FMUs) in South‐East Cameroon. Both park and neighboring forestry concessions require reliable evidence on key factors driving great ape distribution for their management plans, yet this information is largely missing and incomplete. This study aimed at mapping great ape habitat suitability in the area and at identifying the most influential predictors among three predictor categories, including landscape predictors (dense forest, swampy forest, distance to water bodies, and topography), human disturbance predictors (hunting, deforestation, distance to roads, and population density), and bioclimatic predictor (annual precipitation). We found that about 63% of highly to moderately suitable chimpanzee habitat occurred within the Lobéké National Park, while only 8.4% of similar habitat conditions occurred within FMUs. For gorillas, highly and moderately suitable habitats occurred within the Lobéké National Park and its surrounding FMUs (82.6% and 65.5%, respectively). Key determinants of suitable chimpanzee habitat were hunting pressure and dense forest, with species occurrence probability optimal at relatively lower hunting rates and at relatively high‐dense forest areas. Key determinants of suitable gorilla habitat were hunting pressure, dense forests, swampy forests, and slope, with species occurrence probability optimal at relatively high‐dense and swampy forest areas and at areas with mild slopes. Our findings show differential response of the two ape species to forestry activities in the study area, thus aligning with previous studies.

## Graphical abstract

This study aimed at mapping great ape habitat suitability in the Lobéké area and at identifying the most influential predictors that affect species habitat suitability. The results of the study show that about 63% of highly to moderately suitable chimpanzee habitat occurs within the Lobéké National Park, while only 8.4% of similar habitat conditions occur within FMUs. For gorillas, highly and moderately suitable habitats occur within the Lobéké National Park and its surrounding FMUs (82.6% and 65.5%, respectively). Key determinants of suitable chimpanzee habitat were hunting pressure and dense forest, while suitable gorilla habitat was strongly influenced by hunting pressure, dense forests, swampy forests, and slope.

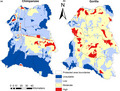

## 1 INTRODUCTION

Chimpanzees and gorillas are large‐bodied primate species frequently occurring at high densities within dense tropical forest and woodland savanna across equatorial Africa (Tutin & Fernandez, 1993). Their large‐scale abundance, distribution, and range make this region of specific interest to great ape conservation.

Chimpanzees are divided into four subspecies: the western chimpanzee (*Pan troglodytes verus*), the central chimpanzee (*Pan troglodytes* *troglodytes*), the eastern chimpanzee (*Pan* *troglodytes* *schweinfurthii*), and the Nigeria–Cameroon chimpanzee (*Pan troglodyte ellioti*). *Pan troglodytes verus* occur within forested areas in West Africa, with a population estimate of approximately 52,800 individuals (Heinicke et al., 2019). *Pan troglodytes* *troglodytes* live along forested areas within Central Africa, with population estimates of approximately 140,000 individuals (Maisels et al., 2016). *Pan* *troglodytes* *schweinfurthii* are found within forested areas in East Africa, with population estimates of about 181,000–256,000 individuals (Plumptre et al., 2016). *Pan troglodyte ellioti* ranges from Cameroon, west of the Sanaga River, to Nigeria, with population estimates of between 6,000 and 9,000 individuals (Morgan et al., 2011; Oates et al., 2016).

Gorillas are divided into the Eastern and Western gorillas, each with two distinct subspecies. They include *Gorilla beringei graueri* and *Gorilla beringei beringei* for the Eastern subspecies (mountain gorillas) and *Gorilla gorilla gorilla* and *Gorilla gorilla diehli* for the Western subspecies (lowland gorillas). *Gorilla beringei beringei* and *Gorilla beringei graueri* inhabit Albertine rift montane forests along the Virunga Mountains of Uganda, Democratic Republic of Congo, and Rwanda with population estimates of approximately 1,000 individuals (Hickey et al., 2018; Roy et al., 2014) and 3,800 individuals (Plumptre et al., 2016), respectively. *Gorilla gorilla gorilla* and *Gorilla gorilla diehli* inhabit dense forests and lowland swamps and marshes of central, west, and east Africa. The former has a population size of approximately 302,973–460,093 individuals (Strindberg et al., 2018) while the latter is estimated at roughly 250–300 individuals, located in forested areas of approximately 12,000 km^2^ (Bergl, 2006; Oates et al., 2003, 2007; Sunderland‐Groves et al., 2003).

In general, the population status of chimpanzees and gorillas is unstable (except for the mountain gorillas). These great apes are facing continuous decline (Brooks et al., 2006; Estrada et al., 2017; Plumptre et al., 2010; Strindberg et al., 2018) mainly due to hunting (e.g., Araújo et al., 2004; Humle et al., 2016; Kuehl et al., 2009; Oates, 1996; Peres & Lake, 2003) and habitat loss (e.g., Chapman & Peres, 2001; Devos et al., 2008; Estrada, 2013; Gippoliti & Dell'Omo, 2003; Humle et al., 2016; Isabirye‐Basuta & Lwanga, 2008; Sá et al., 2012; Yuh et al., 2019). In addition, the occurrence, distribution, and range of chimpanzees also depend upon the extent of forest cover and composition (Yuh et al., 2019), topography (Fitzgerald et al., 2017), climatic variability (Kosheleff & Anderson, 2009; Pruetz, 2007; Reed & Fleagle, 1995), and other human disturbance conditions, for example, increasing human population density (Strindberg et al., 2018), road constructions, and built‐up areas (Estrada et al., 2017). Similar effects are also reported with gorillas (e.g., Estrada et al., 2017; Reed & Fleagle, 1995; Strindberg et al., 2018; Watts, 1988). These factors interact in a complex way to determine great ape habitat suitability across their range (Junker et al., 2012; Plumptre et al., 2010). However, much is still unknown on the difference in spatial variability of suitable chimpanzee and gorilla habitats across various nature reserves where they occur sympatrically. Much is also unknown on how both species respond to the most critical factors that influence their habitat suitability across various nature reserves. Furthermore, the effects of hunting (constituting one of the most important human disturbance factors causing species decline) have not been fully documented in ape distribution or habitat suitability mapping. Thus, mapping and comparing the spatial variability of suitable ape habitats within cohabited reserves, as well as evaluating species response to critical environmental factors, will provide baseline information to aid conservation.

Chimpanzees are highly territorial and as such find most suitable habitats within highly protected areas or National Parks (Heinicke et al., 2019) largely dominated by dense evergreen or swampy forests (Poulsen & Clark, 2004) and with low human disturbance (Stokes et al., 2010; Strindberg et al., 2018). Thus, key determinants of suitable chimpanzee habitats range between dense forested areas, savanna mosaics (Heinicke et al., 2019), and proposed habitat corridors (e.g., distance to built‐up areas such as roads, railways, settlements, etc. (Heinicke et al., 2019; Laurance et al., 2015). Contrary to chimpanzees, gorillas are highly tolerant to forest disturbance and show reduced territoriality, finding most of their suitable habitats within both National parks and certified logging concessions or forest management units largely dominated by swamps or terrestrial herbaceous vegetations (Morgan et al., 2018; Strindberg et al., 2018). Thus, key determinants of suitable gorilla habitats range between swampy forests, grasslands or herbaceous vegetations, and proposed habitat corridors (Strindberg et al., 2018).

Chimpanzees (*Pan troglodytes troglodytes*) and gorillas (*Gorilla gorilla gorilla*) inhabit the Lobéké National Park and its surrounding forest management units (FMUs) in South‐East Cameroon. Both park and forestry concession management require reliable evidence on suitable species habitats (i.e., habitat areas with high species spatial variability), as well as on key factors driving species spatial distribution for their management plans, yet this information is largely missing or incomplete. To address this concern, there is a need to map species habitat suitability and evaluate their response in order to allow conservation to focus on critical species sites (i.e., essential, priority, or highly suitable species sites) while developing long‐term sustainability plans on areas of high human disturbance.

One of the most useful tools that have been widely used in predicting and mapping suitable species habitats is species distribution models (e.g., MaxEnt; Phillips et al., 2006). These models relate sets of species occurrence data to biophysical and environmental factors deemed relevant for predicting species distribution across a given geographic scale. These models have been applied in some primatology studies to map species spatial distribution and habitat suitability across large geographic scales. For example, Junker et al. (2012) have used the MaxEnt species distribution model to predict the recent decline in suitable environmental conditions for African Great Apes. Fitzgerald et al. (2017) have used the MaxEnt model to predict habitat suitability for chimpanzees in the Greater Nimba landscape of Guinea. Plumptre et al. (2010) have used the MaxEnt model to map the occurrence of Eastern chimpanzees and identify suitable areas for establishing surveys.

In this study, therefore, we aimed at (a) using the MaxEnt species distribution model to predict and map chimpanzee and gorilla habitat suitability at the Lobéké National Park and its surrounding forest management units (FMUs), under the influence of environmental and anthropogenic factors that have been shown to affect species distribution; (b) quantifying habitat suitability for both species, and at identifying the most critical factors that influence species habitat suitability; (c) evaluating the differential response of species, and as well, propose measures for species habitat protection and management.

We thus hypothesize that (a) chimpanzees find highly suitable habitats within National Parks while gorillas also find suitable habitats within forest management units; (b) dense forest areas act as key determinants of suitable chimpanzee habitats while suitable gorilla habitats are mostly influenced by swampy forests.

## 2 METHODS

### 2.1 The study area

**FIGURE 1:**
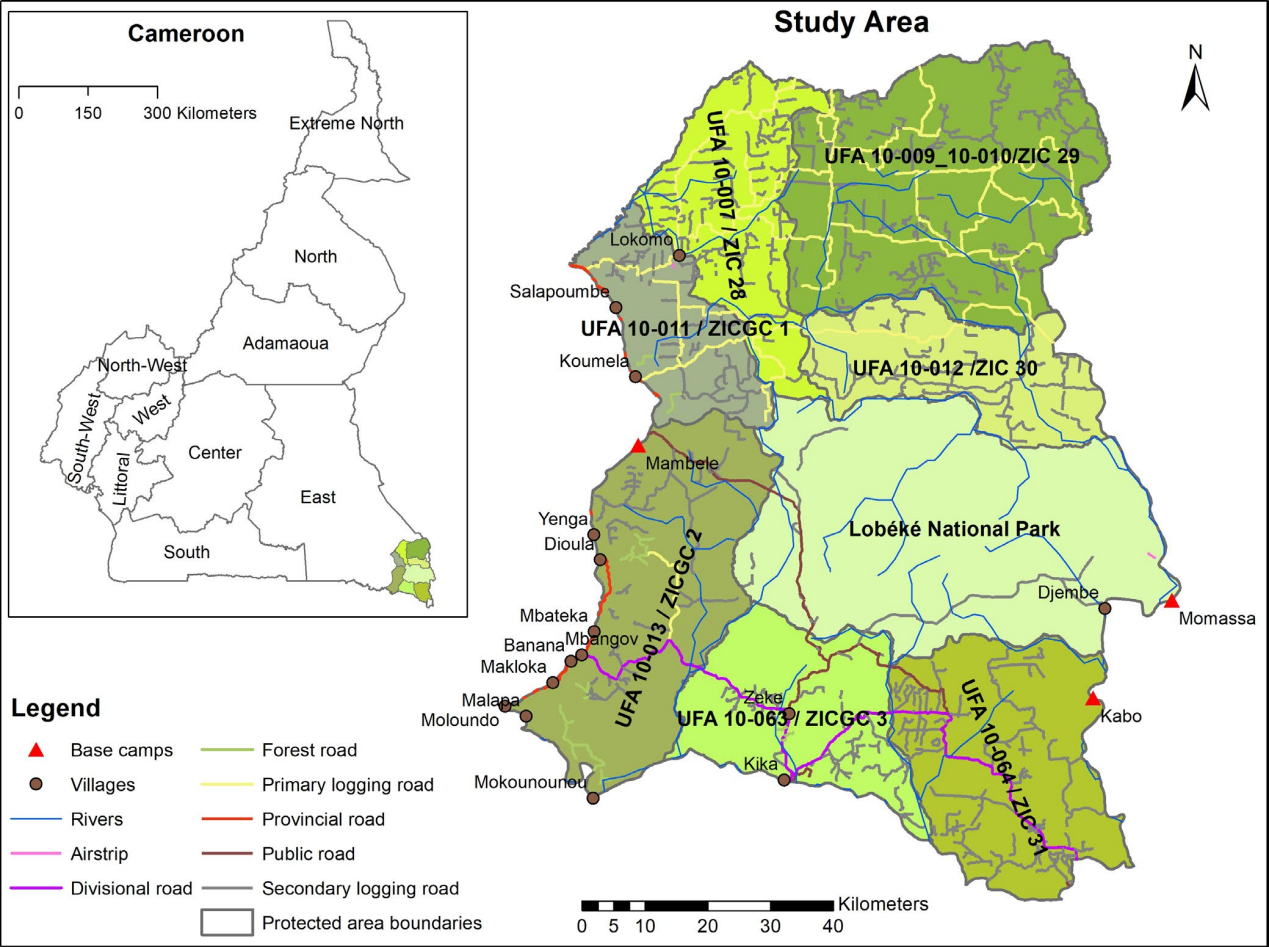
Map of the study area. Map shows the Lobéké National park and its surrounding FMUs

The study area is situated in South‐Eastern Cameroon and covers the Lobéké National Park and its surrounding forest management units (Figure 1). The area lies between latitudes 2°05′ to 2°30′N and longitudes 15°33′ to 16°11′E, with altitudes ranging from 500 to 820 m above sea level. The study site is bounded to the East by the Sanaga river which serves as Cameroon's international border with the Central African Republic and the Republic of Congo. It thus forms part of a trans‐boundary regional protected area network that includes two other National Parks: the Nouabale‐Ndoki National Park in Congo‐Brazzaville and the Dzanga‐Ndoki National Park in the Central African Republic. This network of trans‐boundary protected area is funded by the Central African Forest Commission (COMIFAC) and managed by WWF, GIZ, and WCS Cameroon.

The Lobéké National Park covers a total surface area of 217,334 ha while the FMUs cover total 717,550 ha, divided into seven blocks (Table 1). The national park is a protected area under IUCN category II managed by a conservator from the Ministry of Forestry and Wildlife (MINFOF), Cameroon. The FMUs are logging concessions (but not protected areas) attributed to logging companies for certified timber exploitation and comanaged by local communities, hunting synergies, and the government.

**TABLE 1:** List of protected areas covering the study area

The study area	Protected area	Total surface area (ha)
National Park	Lobéké National Park	217,334
Forest management units or concessions	ZIC 31 or UFA 10–064	115,917
	ZIC 30 or UFA 10–012	74,504
	ZIC 29 or UFA 10–009 and 10–010	177,317
	ZIC 28 or UFA 10–007	81,770
	ZICGC1 or UFA 10–011	55,309
	ZICGC 2 or UFA 10–013	128,541
	ZICGC 3 or UFA 10–063	83,818

Both the national park and FMUs are covered by 3 categories of habitats, including dense or mature primary forests, swampy forests, and grasslands or lowland vegetation (Yuh et al., 2019). Within these habitat categories are found a large variety of plant and animal species. Examples of plant species include *Ceiba pentandra*, *Terminalia superba,* and *Triplochiton pterygota*. Examples of animal species include Chimpanzees, western lowland gorillas, forest elephants, leopards, Buffalos, etc. (Nzooh Dongmo, N'Goran, Ekodeck, et al., 2016).

Bordering the study area to the west are villages inhabited by the Baka community, whose daily activities include commercial hunting, logging, and farming. According to Nzooh Dongmo, N'Goran, Etoga, et al. (2016), hunting rates are significantly high across the entire study area.

Several road types also exist within the study area for easy access by the Baka community and the general public. Road types include forest roads, primary and secondary logging roads, public roads, and provincial and divisional roads.

### 2.2 Data acquisition

#### 2.2.1 Acquisition and preparation of Great Apes data

Presence data for Great Apes (chimpanzee and gorilla) were obtained from the IUCN SSC APES database (http://apes.eva.mpg.de/). The data contain presence points on great ape nests (both fresh and old) collected within the Lobéké National Park and its surrounding FMUs for the years 2001–2005, 2014, and 2015 by a team of WWF biomonitoring experts, using line transect distance sampling (Buckland et al., 1993, 2001; Thomas et al., 2010), following the IUCN best practice guidelines for the survey of great apes (Kühl et al., 2008). Nesting data were most relevant for the study as ape nests are most often used for estimating population size and abundance (e.g., Kühl et al., 2008; Moore & Vigilant, 2013; Pruetz et al., 2002; Strindberg et al., 2018), as well as map suitable habitats to aid conservation (Junker et al., 2012; Plumptre et al., 2010; Strindberg et al., 2018).

In the field sampling approach, a total of eight teams were formulated with each team comprising of 8 field assistants. Within these assistants were GPS and topofil operators, data entry assistants, and decameter operators. GPS and topofil operators made ground observations and measured transect distances. Data entry assistants recorded all ape observations both on the ground and on tree canopies, and decameter operators recorded all human activities and measured perpendicular distances for each observation made. These task distributions aided in avoiding double counting along transect walks.

In general, a total of 1,551 km transect distance were covered during the entire data collection period. From this distance, 288 km was covered at the Lobéké National Park while 1,263 km was covered at the surrounding FMUs. Stratification of the landscapes for data collection was done following Cameroon's decree N ° 0221/MINFOF of 02 May 2006 defining standards for wildlife inventory (Figure S1).

During transect efforts, chimpanzee and gorilla nests were recorded. Other signs were also recorded such as vocalization, feeding remains, feces, footprints, and tracks. Nest sampling was done individually per species so as to avoid overestimation that could arise through group measurements. For each sampled nest, perpendicular distances were measured while nest decay stage or age, including height, type, and number were recorded. Gorilla nests were differentiated from chimpanzee nests through signs of feces, odor, hair, and ground nesting. Ground nesting was more robust for differentiation (Tutin & Fernandez, 1984) as chimpanzees do not build ground nests in this region.

**FIGURE 2:**
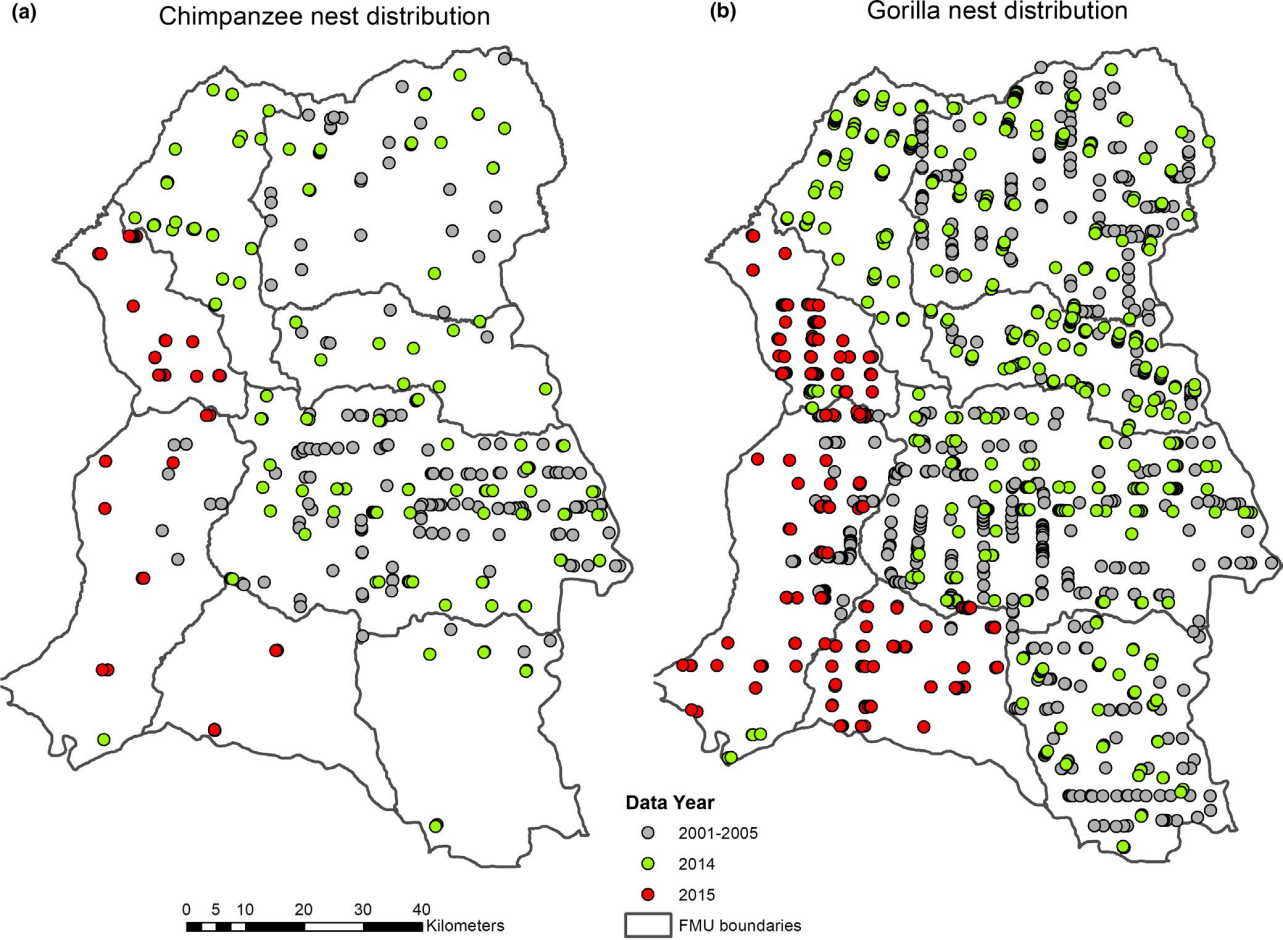
Sample distribution of Great Ape nests for the data collection periods 2001–2006, 2014, and 2015: (a) chimpanzee nest distribution; (b) gorilla nest distribution

We merged all nest datasets in ArcGIS and then established a 1km x1km cell grid within the study area in order to eliminate all duplicated points (nests), thereby reducing sampling bias. From the merged data, we extracted all chimpanzee nests (*N* = 468) separately from gorilla nests (*N* = 1,736). These extractions were based on the different data collection periods. For the periods 2001–2005, *N* = 176 for chimpanzees and *N* = 557 for gorillas, for the years 2014 and 2015, *N* = 236 and *N* = 56 for chimpanzees, and *N* = 872 and *N* = 307 for gorillas. These datasets served as our species occurrence data (presence data) required for habitat suitability modeling (Figure 2).

#### 2.2.2 Acquisition and preparation of environmental and human disturbance factors

To attain our research objectives, we acquired three main predictor categories, divided into 18 predictor variables and obtained from a variety of sources (Table 2). They include human disturbance, landscape, and bioclimatic predictors. Human disturbance predictors comprised mainly of hunting, deforested areas, distance to roads, distance to built‐up areas, and population density (Plumptre et al., 2010; Strindberg et al., 2018; Zhao et al., 2018). Landscape predictors comprised primarily of topography (aspect, slope, and elevation), distance to water bodies, forest cover (dense forest and swampy forest), and grassland vegetation (e.g., Fitzgerald et al., 2017; Yuh et al., 2019). Bioclimatic predictors comprised mainly of mean annual temperature, annual precipitation, maximum temperature of the warmest month, minimum temperature of the coldest month, precipitation of driest month, precipitation of wettest month, seasonal temperature, and seasonal precipitations (Franklin, 2010; Mantyka‐Pringle et al., 2012; Manzoor et al., 2018; Molloy et al., 2013; Phillips et al., 2006). Because of the small study area size (Figure 1) and minimal variation in most bioclimatic predictors (Figure S4), we selected only annual precipitation as the main bioclimatic factor for our analysis, considering that the area showed variation in precipitation rates and that annual rainfall influenced chimpanzee spatial variability in parts of western Cameroon (Sesink Clee et al., 2015). Furthermore, datasets for deforestation, swampy forests, and dense forests were selected for the year 2015. We used these datasets for the year 2015 because studies by Yuh et al., (2019) have shown that the forest covers of Lobéké are still intact (about 93%), with only approximately 7% forest cover loss between the years 2001 and 2015.

**TABLE 2:** Predictor variables required for species habitat suitability modeling

Data category	Data type and measurement units	Source	Moving window scale	Description	Anticipated effect on both species (if important)
Bioclimatic data	Annual precipitations (ml)	https://www.worldclim.org/bioclim	Not applicable	Gridded bioclimatic data for the years 1970–2000 commonly used for sdm	Positive nonlinear effect. Apes may show increased probability of occurrence under increase precipitation
Landscape data	Aspect (degrees)	https://data.humdata.org/dataset/cameroon‐elevation‐model	100,000 ha	DEM acquired for Cameroon, from which aspect and slope were extracted	Intermediate effects. Species may respond negatively or positively to aspect directions
	Slope (m)		Not applicable		Positive nonlinear effects. High and mild slopes may favor species distribution
	Elevation (m)		Not applicable		Positive nonlinear effects. Species occurrence may be high at high elevations
	Distance to water bodies (m)	Normalized differential water index (NDWI) (Figure S2)	100,000 ha	NDWI from which distance to water bodies at 1,000 m was calculated	Positive nonlinear effects. Species might find suitable habitats in close proximity to water bodies
	Dense forest (ha)	Yuh et al. (2019)		Forest cover data obtained from the 2014 land cover data for the study area	Positive nonlinear effect. Dense forest might be most suitable for species distribution
	Swampy forest (ha)				Positive nonlinear effect for gorillas and negative for chimpanzees. Chimpanzees might avoid swampy forests while gorillas might find most suitable habitats within swampy forests
Human footprints data	Hunting pressure (no units. depend on number of hunting points)	http://apes.eva.mpg.de/	100,000 ha	Hunting points collected in the field for the period 2001–2015. They include gun shells, hunter traps, hunter camps, hunter footprints and gunshots	Negative nonlinear effect. Species might find suitable habitats in areas with low hunting rates but gorillas might be tolerant
	Distance to roads (m)	Nzooh Dongmo, N'Goran, Ekodeck, et al. (2016)		Roads extracted from map of the study area from which we measured distance to roads at 1,000 m	Positive linear effect. Species occurrence might increase with increase distance to roads
	Population density (number of persons per square kilometer)	https://sedac.ciesin.columbia.edu/data/set/gpw‐v4‐population‐density‐rev11/data‐download		World population density data for the year 2015 from which the study area population was extracted	Negative linear. Species might avoid areas with high population density but gorillas might be tolerant
	Deforestation (ha)	Yuh et al., (2019)		Land cover map for the year 2015 from which deforested areas were extracted	Negative nonlinear effect. Chimpanzees might completely avoid deforested areas while gorillas might be tolerant

**FIGURE 3:**
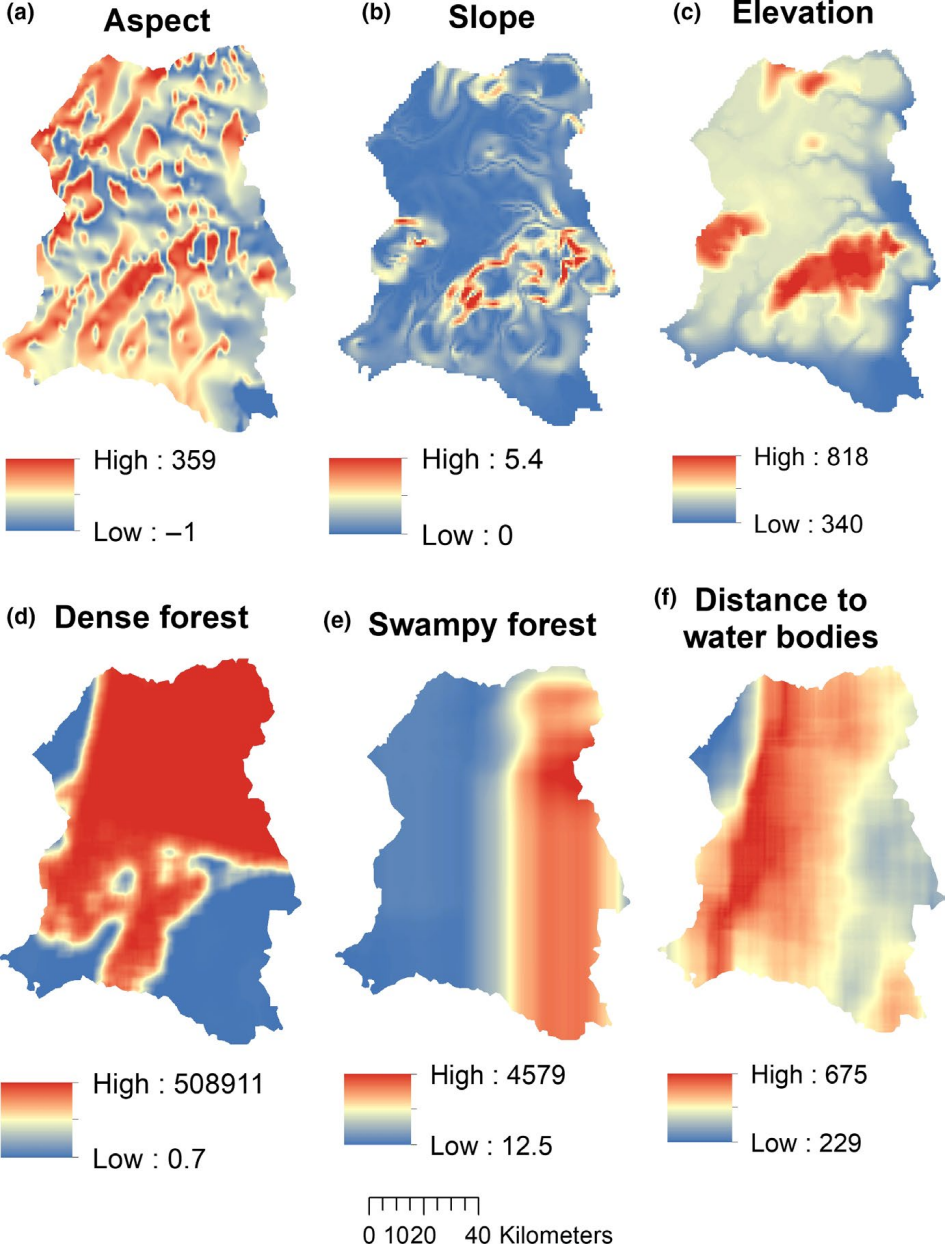
Sample preparation of landscape predictors. Data prepared at a 100,000 ha spatial scale using a neigborhood moving window approach: (a–c) represent topographic variables, that is, (a) aspect; (b) slope; (c) elevation. (d–f) represents land cover variables: (d) dense forests; (e) swampy forests; and (f) distance to water bodies

**FIGURE 4:**
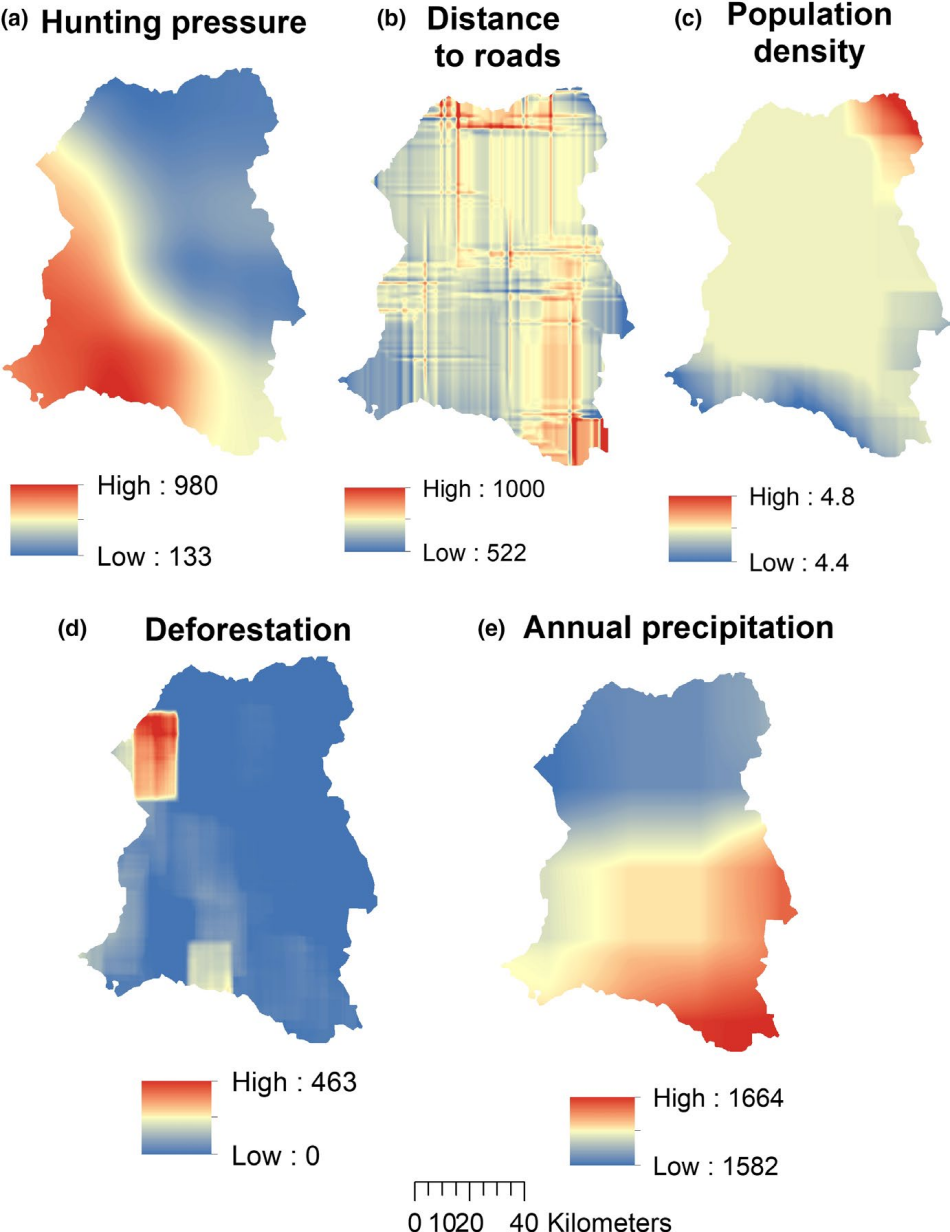
Sample preparation of human disturbance and bioclimatic data. Data prepared at a 100,000 ha spatial scale using a neighborhood moving window approach (except for climate data): (a) hunting pressure; (b) distance to roads; (c) population density; (d) deforestation; (e) annual precipitation

Because we intended to insure spatial independence of all predictor variables, we carried out a Pearson's correlation test in R in order to eliminate strongly correlated datasets so as to insure predictive accuracies and best model performances. We thus found strong correlations between pairs of predictors, that is, between grassland and hunting pressure, and between distance to roads and distance to built‐up areas (Table S1). The criteria for selecting strong correlations were based on *r* values ≥.5. Based on the correlation results, we selected eleven spatially independent predictors for our predictive modeling (Table 2, Figures 3 and 4).

From the three predictor categories in raster format, we interpolated human disturbance and landscape factors at a 500 × 200 m moving window size, by applying the focal statistics tool in ArcGIS (Table 2, Figures 3 and 4). The reason for interpolation at this window size was to predict unknown raster values in areas with no data in each predictor type. All datasets were thus projected to a similar coordinate reference system (WGS 84, UTM zone 33N) and because of different spatial resolutions, we processed them in order they have a similar and fine spatial resolution of 30 m as well as similar spatial extents.

### 2.3 Data analysis

#### 2.3.1 MaxEnt modeling

We applied a maximum entropy (MaxEnt) modeling approach (Phillips et al., 2006) to map the habitat suitability of great apes under the influence of all 11 predictors. MaxEnt is a modeling software designed explicitly for modeling species distribution under a set of gridded environmental conditions and georeferenced occurrence localities. The model predicts explicitly the probability that each pixel within a set of environmental layers contains suitable conditions required for species occurrence. The model presents the advantages of using presence‐only data and performs well with incomplete data, small sample sizes, and gaps (Elith et al., 2006). In the modeling process, therefore, we separately used all chimpanzee and gorilla nest occurrence points (*N* = 468 for chimpanzees and *N* = 1,736 for gorillas) as training datasets and then applied 10,000 background points in each model. We ran the models under 500 iterations, with the program selecting predictors by default with respect to the number of presence points (Phillips et al., 2006). We replicated the model runs ten times and then validated model performances using AUC values determined by the receiver operator characteristics (ROC) (Phillips et al., 2006). The final model outputs were chimpanzee and gorilla habitat suitability maps, species response curves, and contributions of each predictor to species occurrence. The generated suitability maps were further splatted into the eight protected areas covering the study area for comparison purposes (Figures S6 and S7). Furthermore, response curves were analyzed to compare the response of both species to key environmental factors determining species habitat suitability.

#### 2.3.2 Quantifying species habitat suitability

To quantify and compare species habitat suitability thereby evaluating the differential response of species to forest management, we reclassified the generated ape probability maps into four classes of equal intervals in ArcGIS, with probability values ranging from 0 to 1. Probability values ranging between 0 and 0.2 were used to represent unsuitable species habitats; those ranging between 0.2 and 0.4 represented low suitability; 0.4–0.6 represented moderate suitability; and >0.6 represented high suitability (Table S2, Figure S8). The reclassified raster maps were further converted to vector data from which we applied the geometry tool in ArcGIS to quantify and compare suitable and unsuitable areas occupied by both species.

## 3 RESULTS

### 3.1 Model evaluations

**FIGURE 5:**
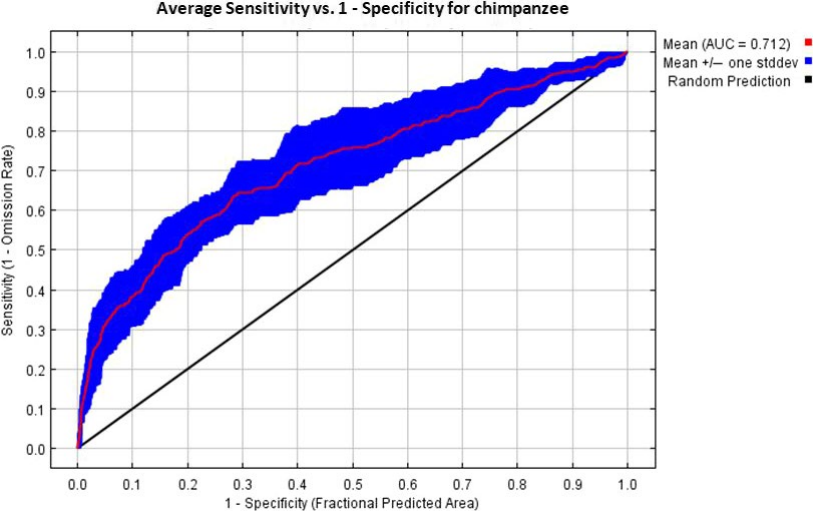
Model evaluation results for chimpanzee predictions

**FIGURE 6:**
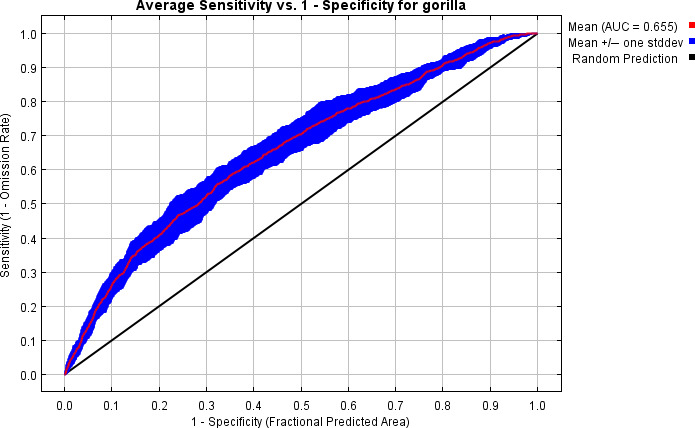
Model evaluation results for gorilla predictions

Our prediction results show that both models performed relatively well (i.e., better than fit) after 10 replicates. For chimpanzee predictions, the average AUC value after 10 replicates was 0.712 (Figure 5), while for gorilla predictions, the average AUC value was 0.655 (Figure 6).

### 3.2 Contributions of predictor variables

The results of the model outputs (Table 3) show that the most important (key factors) that contributed to chimpanzee habitat suitability were dense forests (31.6%) and hunting pressure (22.8%). The total contribution of these two factors summed up to 54.4%. Of the remaining 45.6%, deforestation and distance to water bodies played quite considerable roles, that is, contributed 10.5% and 10.1%, respectively.

**TABLE 3:** Percentage contribution of each predictor variable in determining Great Apes habitat suitability

Variable	Percent contribution for gorilla habitat suitability	Percentage contribution for chimpanzee habitat suitability
Hunting pressure	21	22.8
Dense forest	17.2	31.6
Slope	13.9	3.9
Swampy forest	11.3	2.3
Aspect	9.3	5.2
Annual precipitation	7.2	1.2
Deforestation	6.4	10.5
Elevation	4.9	7.2
Distance to road	4.2	4
Distance to water bodies	3.3	10.1
Population density	1.2	0.6

Key factors that influenced gorilla habitat suitability were hunting pressure (21%), dense forest (17.2%), slope (13.9%), and swampy forests (11.3%). The total contribution of these four factors summed up to 63.4%, while the remaining 36.6% were less important factors.

#### 3.2.1 Effects of single environmental predictors (key factors)

**FIGURE 7:**
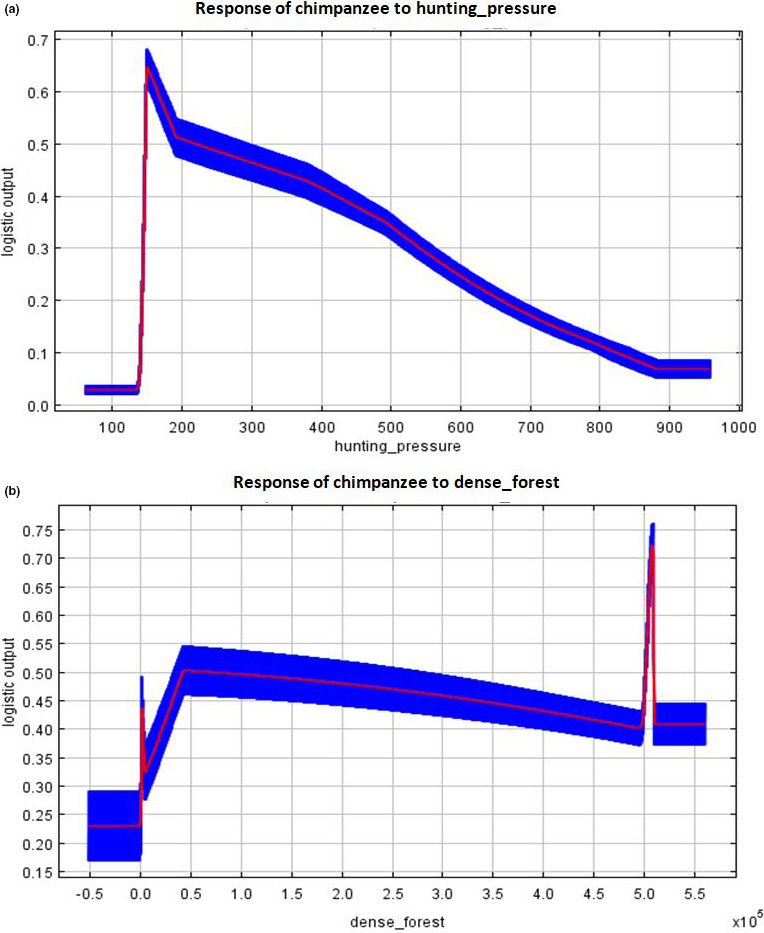
Response of chimpanzees to the most important factors determining habitat suitability: (a) hunting pressure; (b) dense forest

**FIGURE 8:**
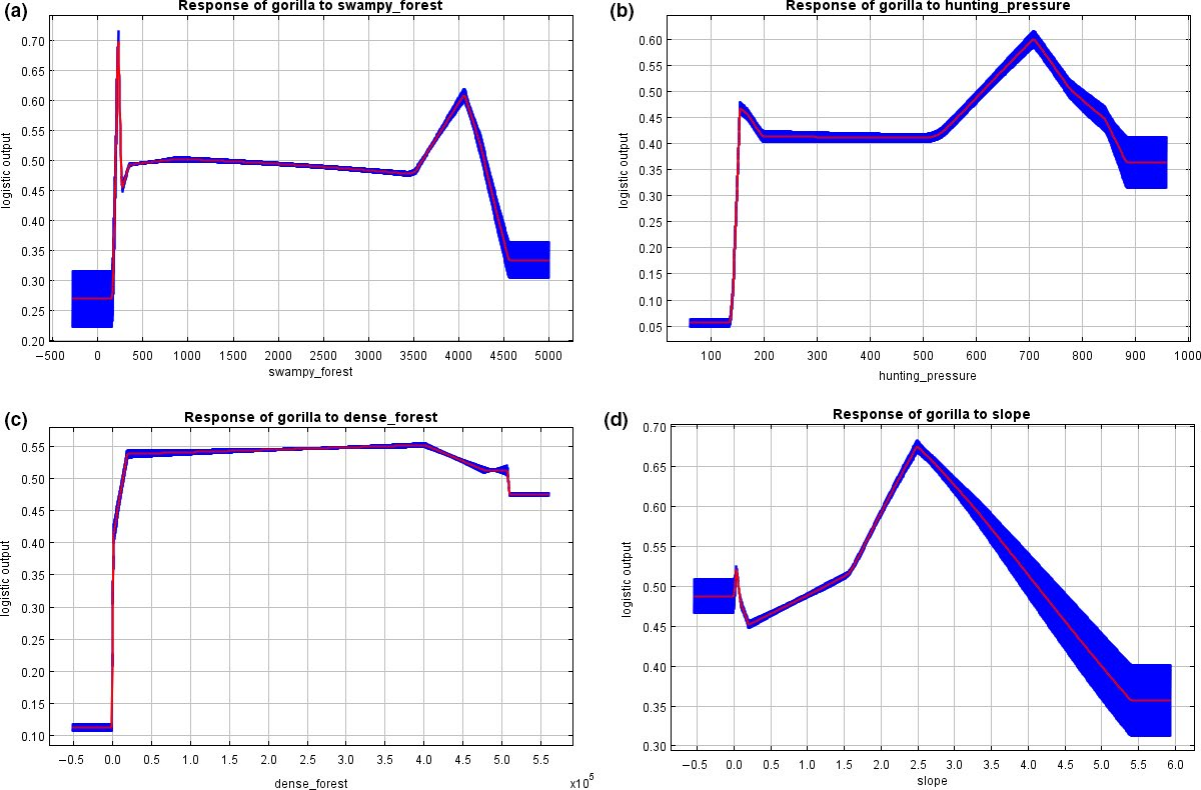
Response of gorillas to the most important factors determining habitat suitability: (a) swampy forest; (b) hunting pressure; (c) dense forest; (d) slope

The variable response curves from the MaxEnt model outputs (Figures 7 and 8) show that the probability of finding suitable chimpanzee habitats was optimal at relatively lower hunting rates (Figure 7a) and at relatively high‐dense forest areas (Figure 7b). This shows that chimpanzees are highly affected by high hunting pressure within the study area but their probability of occurrence increases with increase dense forest areas. With gorillas, they also showed relatively strong response to highly dense and swampy forest areas (Figure 8a,c) but responded differentially to high hunting rates as compared to chimpanzees (Figure 8b). They also show high probability of occurrence or find suitable habitats in areas of mild slopes (Figure 8d).

### 3.3 Mapping and quantification of species habitat suitability

The results of our study show that suitable and moderately suitable chimpanzee habitats cover 11.7% and 51.4% of the entire national park, while low and unsuitable habitats cover 34% and 2.9%, respectively. For the FMUs, high and moderately suitable chimpanzee habitats average 1.4% and 7%, while low and unsuitable habitats average 47.2% and 44.4%, respectively (Figure 10a).

For the entire national park, suitable and moderately suitable gorilla habitats cover 13.4% and 69.2%, respectively, while low and unsuitable habitats cover 17.2% and 0.2%, respectively. For the FMUs, high and moderately suitable gorilla habitats average 8.9% and 56.6%, respectively, while low and unsuitable habitats averaged 26% and 8.6%, respectively (Figure 10b).

## 4 DISCUSSION

### 4.1 Differential response of chimpanzees and gorillas to forest management areas

**FIGURE 9:**
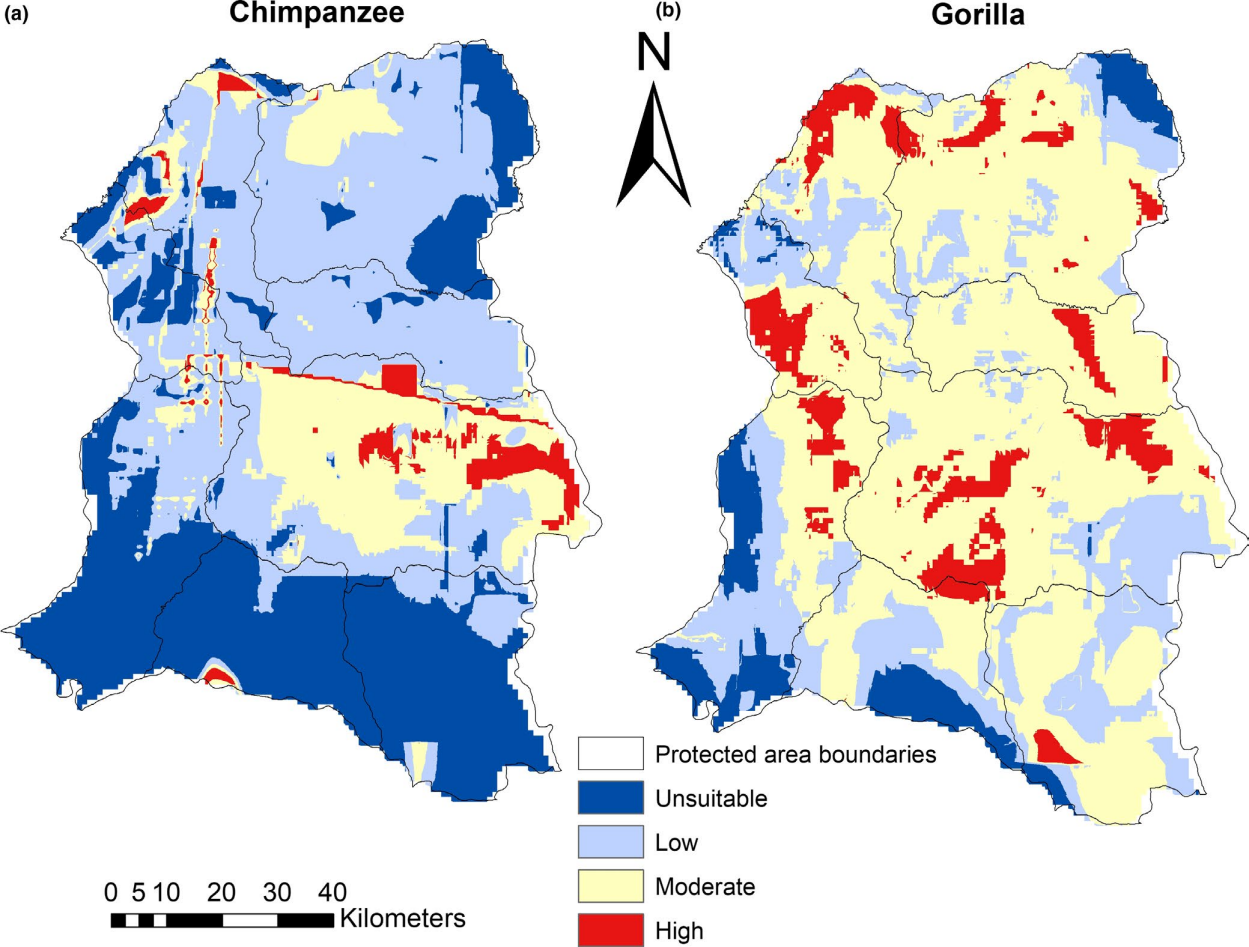
Habitat suitability maps for Great Apes in the entire study area: (a) chimpanzee habitat suitability; (b) gorilla habitat suitability. Maps show species variability within all protected areas with extracts presented in Figures S6 and S7

**FIGURE 10:**
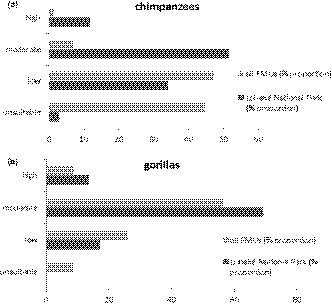
Comparison in chimpanzee and gorilla habitat suitability between the Lobéké National Park and its surrounding FMUs; (a) chimpanzees; (b) gorillas. Figures for all FMUs were derived from average suitability of all FMUs calculated in Table S2

Our findings show that chimpanzees and gorillas respond very differently to forest management in the Lobéké area. Chimpanzees find high and moderately suitable habitats within the Lobéké National Park, while low and unsuitable habitats occur at high proportions within FMUs (Figures 9a and 10a, Figures S6 and S8a). Contrary to chimpanzees, gorillas find high and moderately suitable habitats within both the national park and its surrounding FMUs, with low and unsuitable habitats occurring at low proportions within the entire study area (Figures 9b and 10b, Figures S7 and S8b). Thus, while suitable chimpanzee habitats are significantly more confined to the Lobéké National Park, suitable gorilla habitats are much widely distributed across the study area, including the forestry concessions. This difference in species habitat suitability matches previous patterns of chimpanzee and gorilla population abundance estimates documented by N'Goran et al., (2016), Nzooh Dongmo, N'Goran, Ekodeck, et al. (2016), Nzooh Dongmo et al. (2015) and Nzooh Dongmo, N'Goran, Etoga, et al. (2016). In addition, these findings are in line with previous studies (e.g., Morgan et al., 2018; Strindberg et al., 2018), suggesting a higher tolerance of gorillas to forest disturbance, which can be explained by species benefitting from successional vegetation in the forestry concessions, reduced territoriality, and dependence on ripe fruits compared to chimpanzees.

Chimpanzees are known to be highly territorial and as such have limited abilities to shift spatially to neighboring forestry concessions (Goodall, 1986; Mitani et al., 2010). They are thus more confined or find most suitable habitats within National Parks or highly protected areas with restricted human incursions and high availability of preferred fruits (Bourliere, 1985). With gorillas, their reduced territoriality and high tolerance to forest disturbance make them flexible in finding suitable habitats within both forestry concessions and highly protected areas or National Parks. They are thus capable of occurring or spatially distributed across various forest landscapes or nature reserves but most often settle in areas with high availability of terrestrial herbaceous vegetations (Morgan et al., 2018) and low levels of human impact (Arnhem et al., 2008; Matthews & Matthews, 2004).

### 4.2 Contributions of environmental and human disturbance factors to species habitat suitability

Our study provides broad‐based evidence that the main human disturbance factor that influence habitat suitability of great apes in our study area is hunting pressure (Figure  7a and 8b). Chimpanzees avoid nesting in habitat areas with high hunting pressure. Their probability of occurrence thus decreased under increase hunting pressure (Figure 7a). Beyond a certain threshold, hunting showed less of an impact on gorillas (Figure 8b). They mostly nest in hilly or sloppy areas that are less accessible to hunters. They have equally been reported to be very aggressive toward hunters, usually ambushing them unexpectedly (Köhler, 2005). In general, large mammal hunting has been reported to be the primary source of income generation to the Baka community inhabiting the study area (N'Goran et al., 2016; Nzooh Dongmo, N'Goran, Ekodeck, et al., 2016; Nzooh Dongmo et al., 2015; Nzooh Dongmo, N'Goran, Etoga, et al., 2016). These community inhabitants hunt more within FMUs as compared to the Lobéké National Park, possibly due to strict restrictions on human incursions within the park. Thus, hunting rates at the national park are estimated at 0.25 hunting points/km as compared to an average rate of 0.52 hunting points/km within FMUs (Nzooh Dongmo, N'Goran, Ekodeck, et al., 2016). However, chimpanzees and gorillas are not primary hunting targets (Nzooh Dongmo, N'Goran, Ekodeck, et al., 2016). Reports by Duda et al., (2017), Duda et al., (2018) have shown that the Baka community do not consume great apes meat even though not a strict social norm, particularly on chimpanzees whose market demands are quite high. These local community inhabitants belief species are closely related to humans and are capable of using tools and performing other social cognitive behaviors, whose probability of decline may be likely if consistently hunted. Gorillas in particular are less hunted even though they are sometimes killed opportunistically, a phenomenon that has been reported in many Western African Countries (Bennett, 2007; Fa & Brown, 2009; Tutin, 2001; Vanthomme et al., 2010). Chimpanzees are more targeted than gorillas, particularly within FMUs where hunting rates are relatively high (Nzooh Dongmo, N'Goran, Ekodeck, et al., 2016). This may explain their significantly lower habitat suitability within FMUs.

Our findings further reveal that the main landscape factor that supports chimpanzee habitat suitability is dense forest, while for gorillas, factors include dense forests, swampy forests, and slopes. Dense forests cover over 58% of the entire study area, while swampy forests represent about 40%, totaling 98% forest cover (Yuh et al., 2019). The forests of this area are therefore entirely intact and are thus highly suitable for both species. Chimpanzees are more likely to occur within undisturbed areas (Johns & Skorupa, 1987; Strindberg et al., 2018; Tutin & Fernandez, 1984; White, 1992), making the Lobéké National Park a highly suitable habitat for them as compared to the FMUs. Gorillas on the other hand prefer habitat types composed mainly of terrestrial herbaceous vegetation alongside mature secondary forests (Carroll, 1988; Fay, 1997), especially when under strict hunting regulations and antipoaching controls (Clark et al., 2009; Matthews & Matthews, 2004; Morgan & Sanz, 2007; Stokes et al., 2010; Walsh et al., 2008; Wright, 2003). They thus occur at high densities within protected areas and certified logging concessions largely dominated by swamps (Strindberg et al., 2018), a reason for their high suitability within both the Lobéké National Park and its bounded FMUs. In general, conservation landscapes dominated mainly by dense and swampy forests, and surrounded primarily by protected areas and certified FMUs often maximize suitable wildlife habitats (IUCN, 2014; Mackinnon et al., 2016). With chimpanzees and gorillas, they find highly suitable habitats within such areas in order to benefit from preferred fruit types, as well as avoid interaction with humans. Furthermore, mild slopes seem to favor gorilla movements as such slope conditions favor nest building (Groves & Pi, 1985). Because gorillas mostly build ground nests, they seem to find suitable habitats in sloped landscapes as such landscapes are difficult to access by humans, who might kill them opportunistically. Hilly slopes thus provide refuge to gorillas from hunting as reported in some parts of Cross River region of Nigeria and Cameroon (Bergl & Vigilant, 2007; Morgan et al., 2003; Oates et al., 2003).

Because our study area consists of intact forest habitats (Yuh et al., 2019) due to continuous protected area monitoring, the effects of deforestation, distance to roads, and population density were less important. Logging activities are strictly prohibited in Lobéké. Thus, the creation of new roads for easy access to logging and building of loggers camps is limited, particularly within the Lobéké National Park where access is entirely restricted. Thus, the main roads and buildings that exist in the area include old logging roads and camps created by hunters and easily accessed by gorillas (Laurance, 2006). Because of such limitations, human population density within the study area is extremely low. These conditions thus favor the distribution of species, particularly gorillas who find suitable habitats within the entire study area. Chimpanzees find large extents of suitable habitats within the National Park as this area is undisturbed, rich in available fruit types, and completely restricted. Their low suitability within FMUs indicates species are highly threatened within these zones, with effects likely due to high hunting pressure and less availability of preferred fruit trees.

The contribution of other landscape features such as aspect, elevation, and distance to water bodies was less important predictors of chimpanzee and gorilla occurrence. The amount of water bodies found within Lobéké is relatively small (Yuh et al., 2019). The landscape is also moderately elevated. These conditions combined, seemed less significant in predicting species habitat suitability. Another less important factor was annual precipitation. The low predictability of this bioclimatic factor could only imply species are less sensitive to such bioclimatic condition, probably due to forest shading, despite the high spatial variability in precipitation rates.

### 4.3 Limitations of the study

Although our findings provide reasonable evidence on suitable species habitats, as well as on the key factors affecting species habitat suitability, our modeling and quantification approach provide only rough estimates of the true spatial distribution of chimpanzees and gorillas within the entire study area. The development of our model was limited by the quality and spatial variability of our predictor datasets. For example, with bioclimatic factors, we used only annual precipitations as this was the only factor that provided a good spatial variability in climatic conditions. All other bioclimatic variables showed very little or no climate variability and as such, could cause erroneous relationships in our models. Furthermore, the aspect of strong correlations between some environmental factors (Table S1) enabled us to avoid the effects of overlapping variables by dropping some predictors which have been often used in mapping species habitat suitability, for example, built‐up area (Estrada et al., 2017). Furthermore, our models did not consider other factors such as disease (Bermejo et al., 2006; Leendertz et al., 2004; Walsh et al., 2003), presence–absence of guards, food availability (Morgan et al., 2018), and other conservation activities aimed at reducing human impacts (Tranquilli et al., 2011). Finally, our response variable incorporated only chimpanzee and gorilla nests signs which might not always reflect area usage, though robust in species habitat suitability mapping. Thus, other variables (such as tool use sites) which could have been used as responds variables to improve suitability mapping were not considered. Such weakness was obviously due to the unavailability of existing datasets.

Based on the above limitations, we consider our model estimates as the first attempt in providing evidence on suitable species habitats within the Lobéké National Park and its surrounding FMUs, as well as on key factors driving species spatial distribution to aid conservation within the entire study area. We thus recommend further work to be done to improve our models.

## 5 CONCLUSION

We used the MaxEnt model to predict, map, and quantify chimpanzee and gorilla habitat suitability within Lobéké, influenced by three predictor categories (landscape, human disturbance, and bioclimatic predictors). We identified single predictors that were most important in predicting species habitat suitability in our study area. For chimpanzees, these include hunting pressure (human disturbance) and dense forests, while for gorillas, these include hunting pressure (human disturbance), dense forest, swampy forest, and slopes (Landscape). The high contributions of these factors enabled us to conclude that large proportions of suitable chimpanzee habitats occur within the Lobéké National Park while species face severe threats within FMUs. In addition, FMUs do not provide attractive resources for chimpanzees. Chimpanzees thus need serious attention. For gorillas, their high suitability is spatially represented within the entire study area. Hence, they are less threatened than chimpanzees but need continuous attention to insure sustainability.

Based on our findings, we propose that a landscape zonation plan be implemented by WWF and the Cameroon Ministry of Forestry and wildlife to separate suitable and moderately suitable species habitats from low and unsuitable species sites. This zonation plan will aid continuous monitoring to sustain critical species habitats while providing basis for upgrading moderately suitable habitats to high suitability. This plan will equally define areas ideal for future surveys.

We also propose strengthening legislation and sensitization campaigns to continuously reduce hunting pressure, and educate on the need to protect critical species habitats and upgrade moderately suitable sites. We also propose revising and upgrading laws governing the use of forest concessions. This will help in gradually recovering low and unsuitable species sites in the long run. The zonation plan, legislation, and education programs will equally benefit other wildlife or large mammal species inhabiting the entire study area such as forest elephants, leopards, buffalos, etc.

## CONFLICT OF INTEREST

The authors declare no competing financial interest.

## AUTHOR CONTRIBUTIONS

**Yisa Ginath Yuh:** Conceptualization (lead); Formal analysis (lead); Investigation (lead); Methodology (lead); Software (lead); Validation (lead); Visualization (lead); Writing‐original draft (lead); Writing‐review & editing (lead). **Paul K. N'Goran:** Data curation (lead); Investigation (equal); Project administration (lead); Resources (equal); Writing‐review & editing (equal). **Zacharie N Dongmo:** Data curation (lead); Investigation (lead); Project administration (lead); Resources (equal). **Wiktor Tracz:** Conceptualization (equal); Formal analysis (equal); Methodology (equal); Software (supporting); Supervision (lead); Validation (equal); Visualization (equal); Writing‐original draft (supporting); Writing‐review & editing (equal). **Elvis Tangwa:** Formal analysis (supporting); Methodology (supporting); Software (supporting); Validation (equal); Writing‐original draft (supporting); Writing‐review & editing (supporting). **Michael Bode Agunbiade:** Methodology (supporting); Software (supporting); Writing‐review & editing (supporting). **Tenekwetche Sop:** Conceptualization (equal); Data curation (equal); Resources (equal); Validation (equal); Visualization (equal); Writing‐original draft (supporting); Writing‐review & editing (supporting). **Chefor Fortang:** Data curation (equal); Writing‐review & editing (supporting). **Hjalmar Kuehl:** Conceptualization (equal); Data curation (equal); Methodology (equal); Resources (equal); Software (supporting); Supervision (equal); Validation (equal); Visualization (equal); Writing‐review & editing (supporting).

## ETHICAL APPROVAL

The data collection methods for this study were strictly noninvasive and were approved by the Ethical Board of the WWF Cameroon country office. As such, the data were collected in accordance with Cameroon's laws and regulations governing animal research. WWF Cameroon works in partnership with the Ministry of forestry and Wildlife in Cameroon and, as such, has all the necessary permission and MOUs (memorandum of understandings) for data collection within protected areas in Cameroon. As part of the corresponding Authors Masters research work, field data were obtained from the IUCN Apes database at the Max Planck Institute for Evolutionary Anthropology under approval from the WWF Cameroon country office. The available datasets are strictly not for sharing according to the WWF guides and regulations for data availability. Data are, however, available from the Author upon permission from the data providers (WWF Cameroon country office) or can be obtained directly from the A.P.E.S database (http://apes.eva.mpg.de/).

## Supporting information

Supplementary Material

Supplementary Material

## Data Availability

The available ape datasets including GIS raster layers have been made publicly available in Figshare with a DOI accession number provided: 10.6084/m9.figshare.13186532. Detail survey files can also be obtained directly from the IUCN A.P.E.S database (http://apes.eva.mpg.de/).
