# Supplementary material for: Mapping suitable great ape habitat in and around the Lobéké National Park, South‐East Cameroon
Source: Ecol Evol. 2020 Dec 5;10(24):14282–99. doi: 10.1002/ece3.7027 (PMC7771158; doi:10.1002/ece3.7027)
Supplement: Supplementary file 1 — Supplementary Material [file ECE3-10-14282-s001.docx]

**Mapping Suitable Great Ape habitat in and around the Lobéké National Park, South-East Cameroon**

**Supplementary materials**


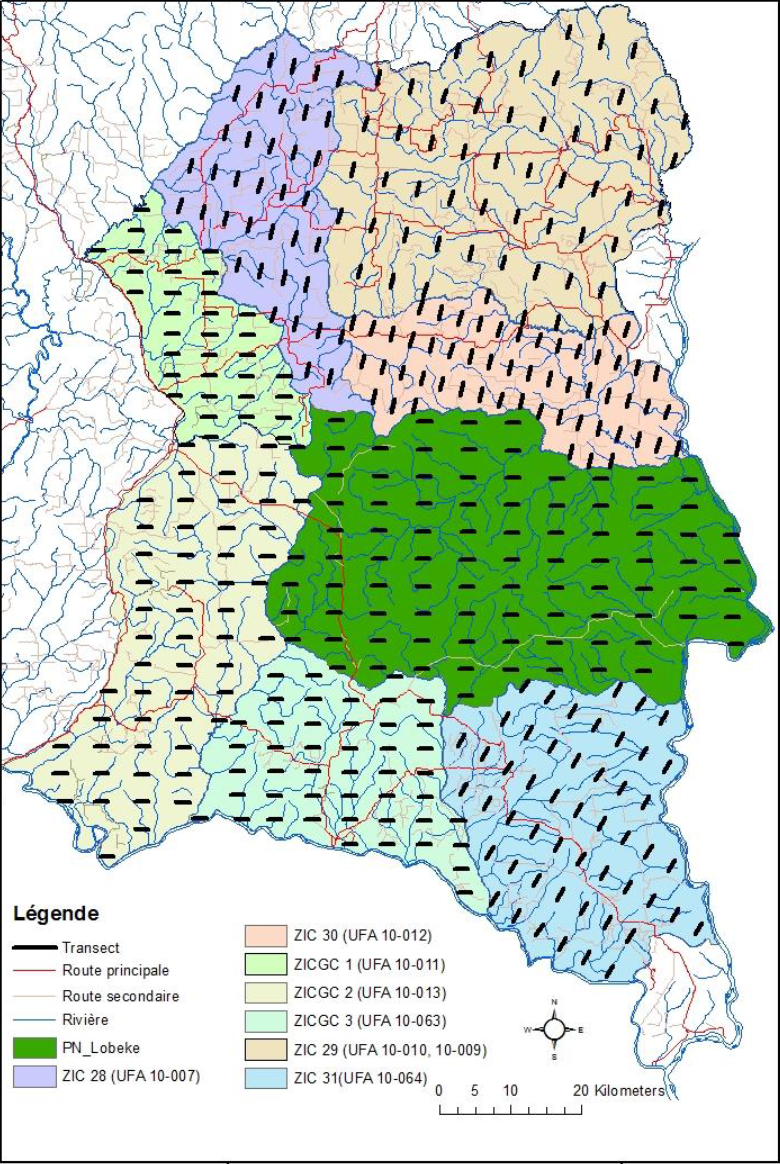


**Figure S1**. Sampling map showing the location of transects in the study area (Map generated by ZacharieNzoo using ArcGIS 10.3.1, referenced in Yuh et al. 2019). Forest management units are represented by: UFA 10-064 / ZIC 31, UFA 10-012 /ZIC 30, UFA 10-009 et 10-010 / ZIC 29, UFA 10-007 / ZIC 28, UFA 10-011 / ZICGC 1, UFA 10-013 / ZICGC 2, UFA 10-063 / ZICGC 3, and the Lobéké National Park (Figure S6). The determination of the macro-zone sounding effort was made by combination of national standards for wildlife inventories in forest areas (decree N ° 0221 / MINFOF of May 02, 2006) and international statistical principles based on the rate meeting the presence indices of the target species (great ape nests).

**
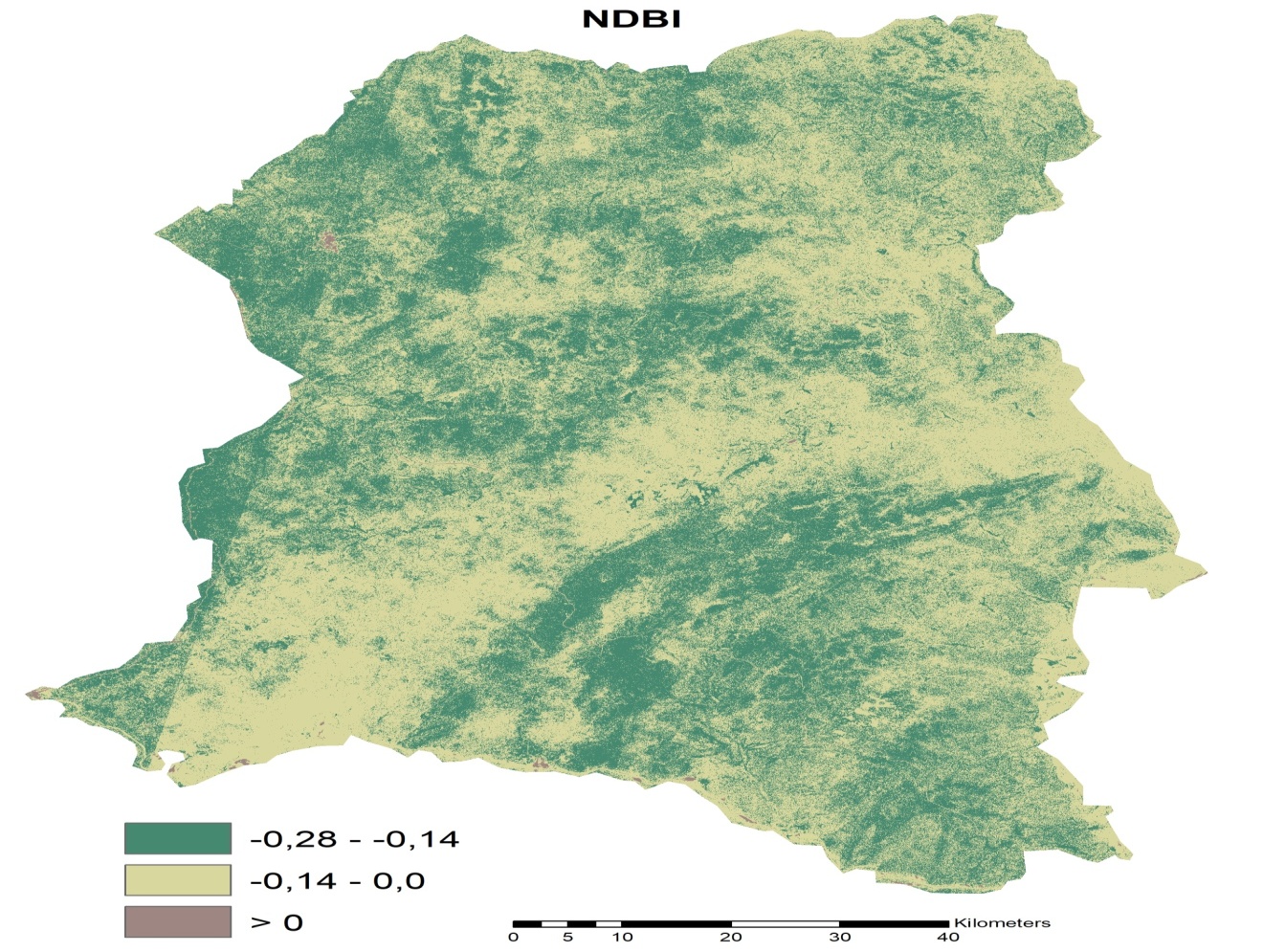
**

**Figure S2.** Normalized Differential Built-up Index. Data used for extracting built up areas (i.e. all areas > 0)

Data on built-up areas were calculated using the Normalized Differential Built-up Index (NDBI) in ArcGIS (equation 1) (Figure S6). We calculated NDBI using the near-infrared (NIR) and short wave infrared (SWIR) bands of Landsat 8 images (Zha et al., 2003; Macarof & Statescu, 2017) of the study area, with acquisition date of 7^th^ January 2015. Because the calculated NDBI values also ranged between -1 and 1, we extracted all areas > 0 to represent built-up areas. From the extracted data, we calculated distance to built-up areas at 1000m units using the Euclidean distance tool in ArcGIS.

NDBI = (SWIR – NIR) / (SWIR + NIR) (1)

**
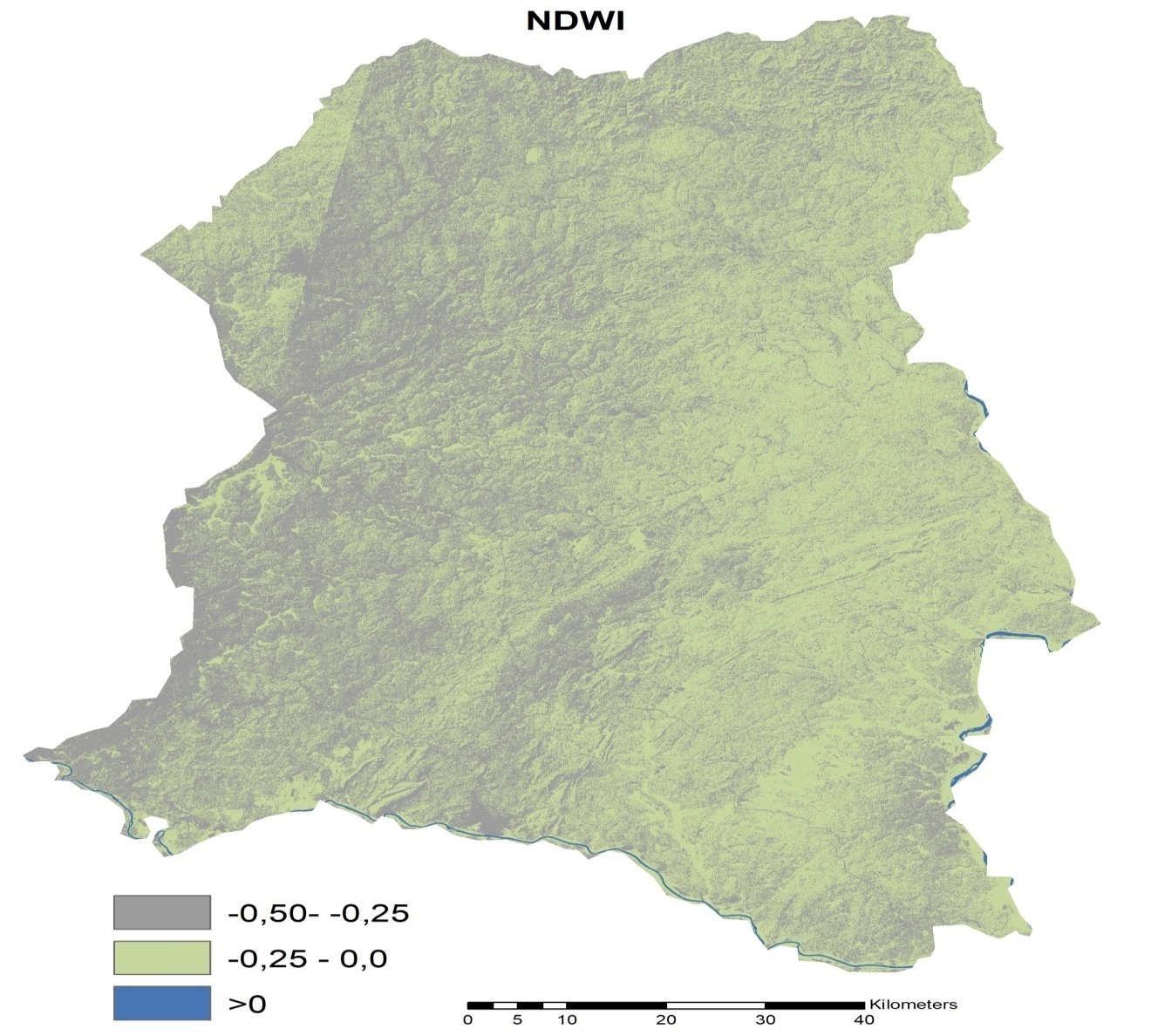
**

**Figure S3.** Normalized Differential Water Index. Data used for extracting water bodies (i.e. all areas > 0)

Data for the presence of water bodies were obtained through the normalized differential water index (NDWI). NDWI was calculated in ArcGIS using the green and SWIR (short wave infra-red) bands of Landsat 8 images (Xu, 2006, equation 2) of the study area, with an acquisition date of 7^th^ January 2015 (Figure S7). We used Landsat 8 images because they have narrower spectral bands with higher 12-bit radiometric resolutions, and more precise geometries (Irons et al., 2012). Because the calculated NDWI values ranged between -1 and 1, we extracted all areas > 0 to represent areas of water presence. From the extracted data, we calculated distance to water bodies at 1000m units using the Euclidean distance tool in ArcGIS.

NDWI = (Green – SWIR) / (Green + SWIR) (2)

**
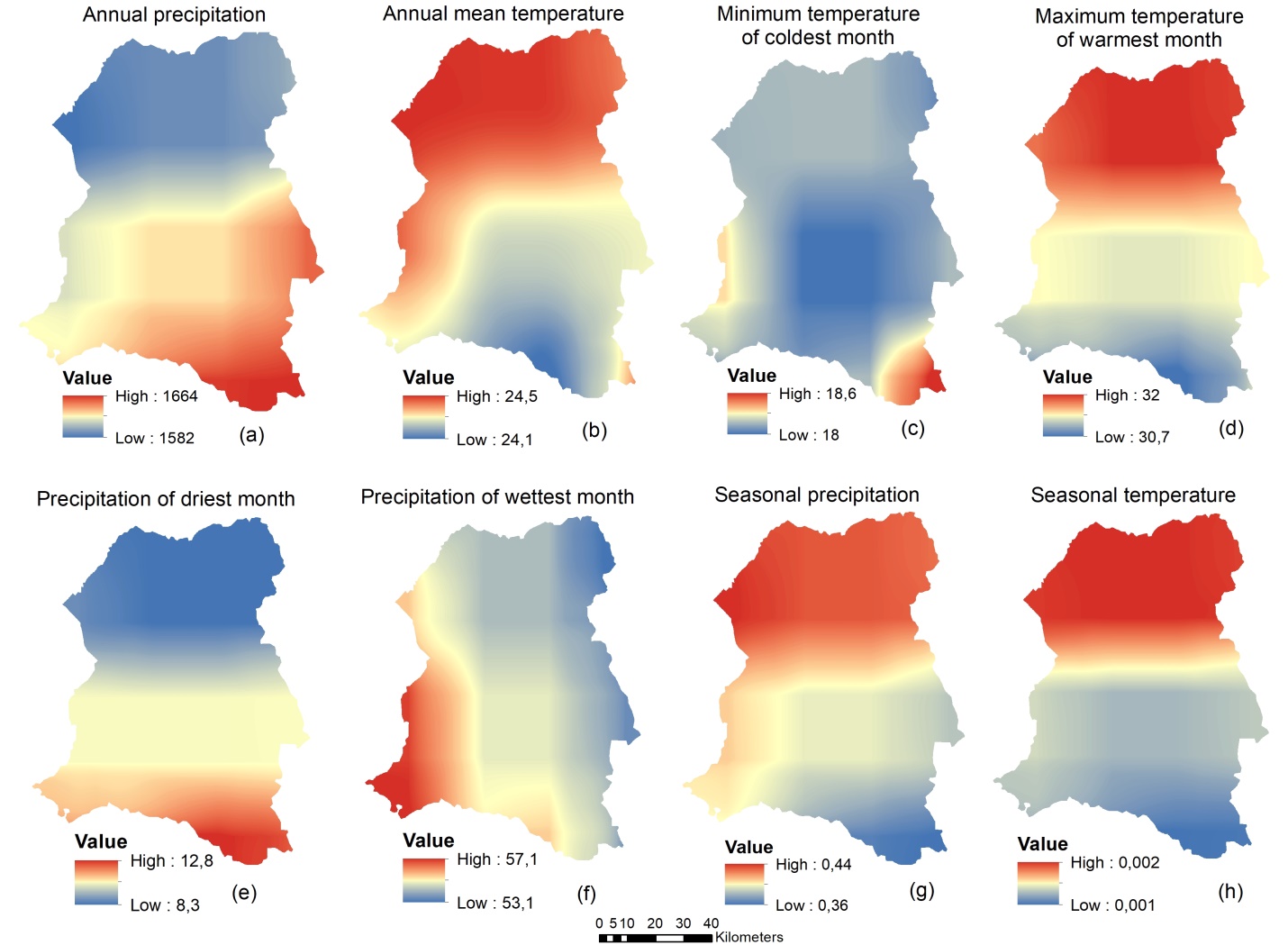
**

**Figures S4.** Sample preparation of bioclimatic variables for the study area: (a) annual precipitation; (b) annual mean temperature; (c) minimum temperature of coldest month; (d) maximum temperature of warmest month; (e) precipitation of driest month; (f) precipitation of warmest month; (g) seasonal precipitation; (h) seasonal temperature


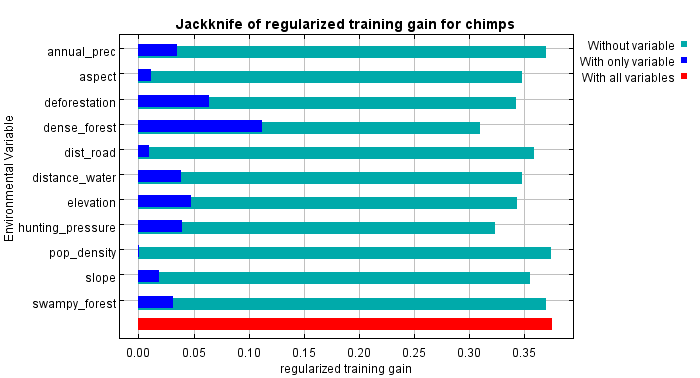


(a)


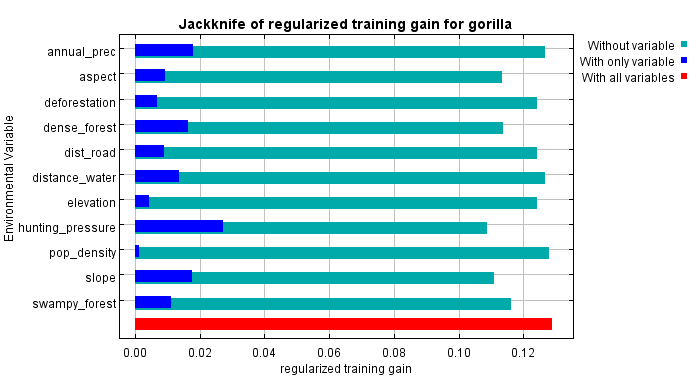


(b)

**Figures S5.** Jacknife of regularized training gains for Great Apes vs environmental variables: (a) gorillas; (b) chimpanzees. Results generated from the MaxEnt model.

**References**

Irons, J. R., Dwyer, J. L., & Barsi, J. A. (2012). The next Landsat satellite: The Landsat data continuity mission. *Remote Sensing of Environment*, **122**, 11–21.

Macarof, P., & Statescu, F. (2017). Comparasion of ndbi and ndvi as indicators of surface urban heat island effect in landsat 8 imagery: A case study of iasi. *Present Environmental and Sustainaible Development,* **11**, 141–150.

Xu, H. (2006). Modification of normalised difference water index (NDWI) to enhance open water features in remotely sensed imagery. *International Journal of Remote Sensing,* **27**, 3025-3033.

Zha, Y., Gao, J., & Ni, S. (2003). Use of normalized difference built-up index in automatically mapping urban areas from tm imagery. *International Journal of Remote Sensing,* **24**, 583–594.
