# Supplementary material for: Mapping suitable great ape habitat in and around the Lobéké National Park, South‐East Cameroon
Source: Ecol Evol. 2020 Dec 5;10(24):14282–99. doi: 10.1002/ece3.7027 (PMC7771158; doi:10.1002/ece3.7027)
Supplement: Supplementary file 2 — Supplementary Material [file ECE3-10-14282-s002.docx]

# Appendices

**Table S1.** Correlation between sets of predictors (strong correlated values are highlighted in light black)

|  | S | AP | A | PD | DF | SF | DW | E | HP | Def | DR | DBU | GR |
| --- | --- | --- | --- | --- | --- | --- | --- | --- | --- | --- | --- | --- | --- |
| S | 1 |  |  |  |  |  |  |  |  |  |  |  |  |
| AP | 0.18 | 1 |  |  |  |  |  |  |  |  |  |  |  |
| A | 0.09 | -0.15 | 1 |  |  |  |  |  |  |  |  |  |  |
| PD | 0.02 | -0.26 | -0.03 | 1 |  |  |  |  |  |  |  |  |  |
| DF | -0.01 | -0.61 | -0.02 | 0.25 | 1 |  |  |  |  |  |  |  |  |
| SF | 0.13 | 0.36 | -0.29 | 0.08 | -0.22 | 1 |  |  |  |  |  |  |  |
| DW | -0.09 | -0.19 | 0.04 | 0.08 | 0.44 | -0.5 | 1 |  |  |  |  |  |  |
| E | 0.4 | -0.2 | 0.22 | 0.15 | 0.24 | -0.19 | 0.19 | 1 |  |  |  |  |  |
| HP | -0.08 | 0.33 | 0.15 | -0.22 | -0.29 | -0.51 | 0.22 | -0.04 | 1 |  |  |  |  |
| Def | -0.02 | -0.01 | 0.01 | -0.02 | -0.03 | -0.01 | -0.03 | -0.02 | 0.02 |  |  |  |  |
| DR | 0.04 | -0.55 | 0.1 | 0.2 | 0.44 | -0.26 | 0.25 | 0.42 | -0.26 | 0 | 1 |  |  |
| DBU | 0.03 | -0.55 | 0.09 | 0.2 | 0.45 | -0.22 | 0.26 | 0.42 | -0.31 | 0 | 0.9 | 1 |  |
| Gr | -0.04 | 0.2 | -0.01 | -0.17 | -0.31 | -0.25 | 0 | -0.16 | 0.55 | -0.01 | -0.32 | -0.36 | 1 |

*AP=annual precipitations, PD=population density, HP=hunting pressure, A=aspect, S=slope, E=elevation, DW=distance to water bodies, DF=dense forest, SF=swampy forest, Def=deforestation, DR=distance to roads, DBU=distance to built-up, GR=grasslands.


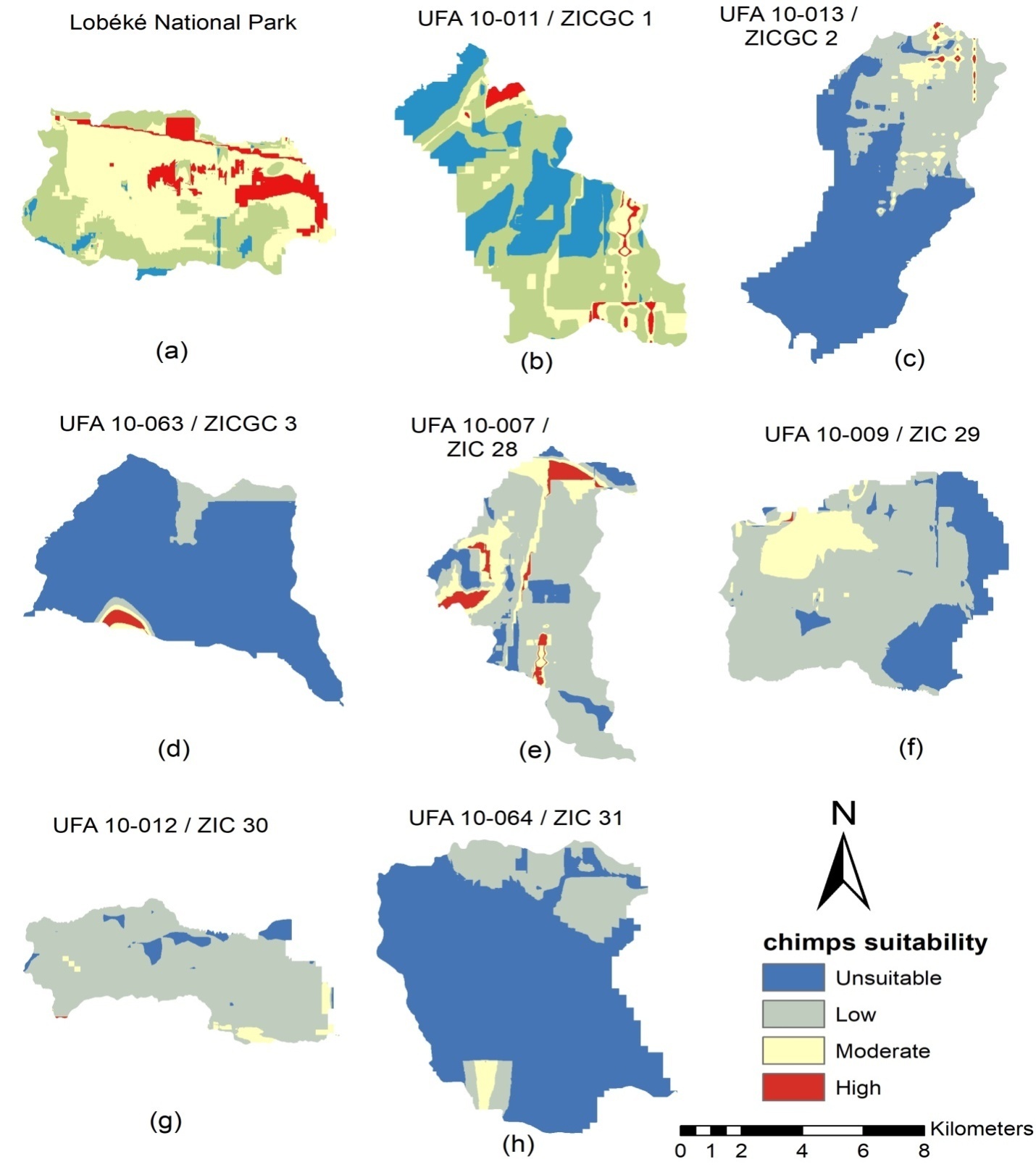


**Figures S6**. Habitat suitability maps for chimpanzees within protected areas: (a) Lobéké National Park; (b) ZICGC1 or UFA 10–007; (c) ZICGC 2 or UFA 10–013; (d) UFA 10–063 or ZICGC 3; (e) ZIC 28 or UFA 10–007; (f) ZIC 29 or UFA 10–009; (g) ZIC 30 or UFA 10–012; (h) ZIC 31 or UFA 10–064


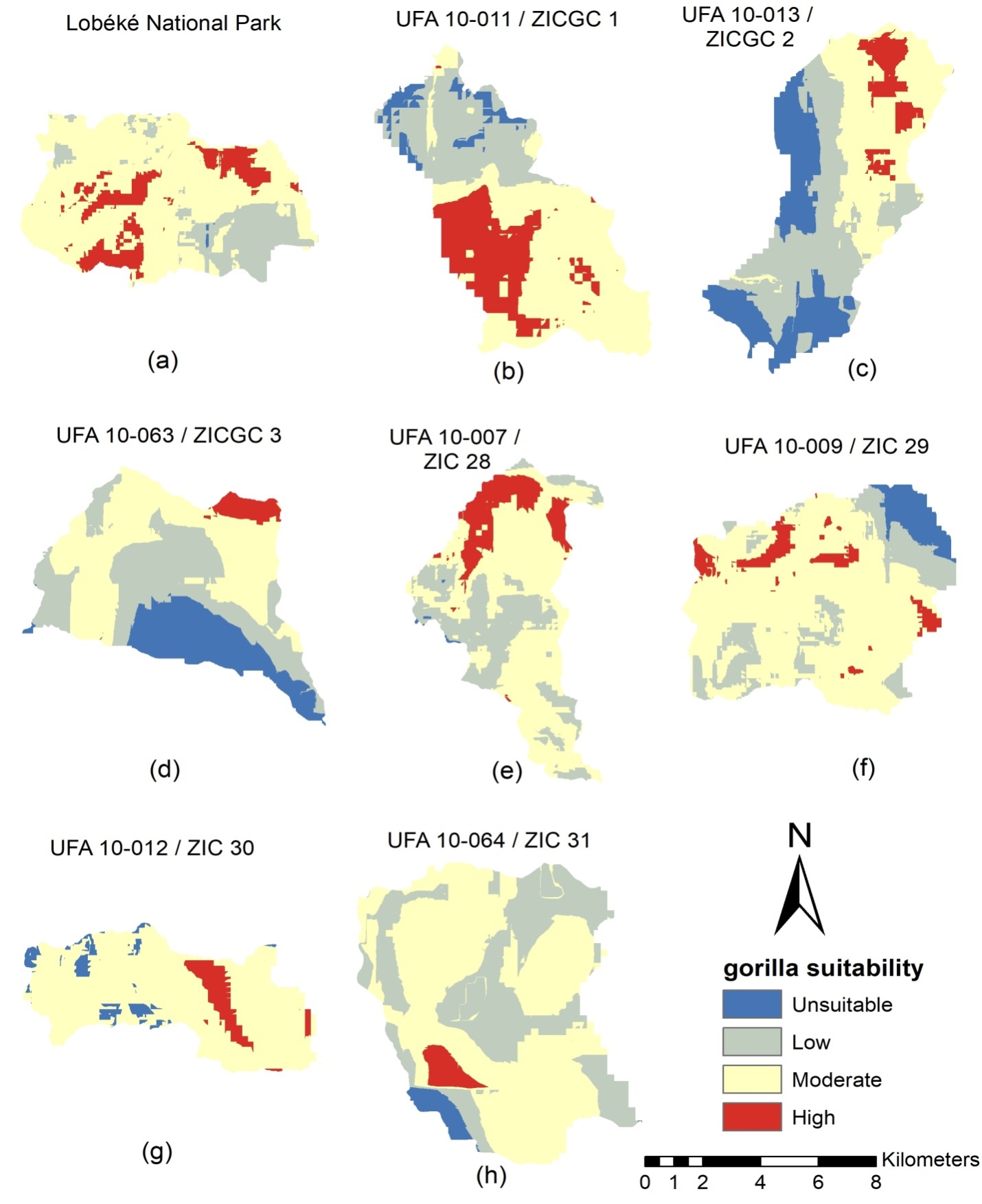


**Figures S7**. Habitat suitability maps for gorillas within protected areas: (a) Lobéké National Park; (b) ZICGC1 or UFA 10–007; (c) ZICGC 2 or UFA 10–013; (d) UFA 10–063 or ZICGC 3; (e) ZIC 28 or UFA 10–007; (f) ZIC 29 or UFA 10–009; (g) ZIC 30 or UFA 10–012; (h) ZIC 31 or UFA 10–064

**Table S2.** Percentage habitat suitability and area for chimpanzees and gorillas within protected areas

| Species | Class | Lobéké National Park | | ZICGC1 or UFA 10–007 | | ZICGC2 or UFA 10–013 | | UFA 10–063 or ZICGC 3 | |
| --- | --- | --- | --- | --- | --- | --- | --- | --- | --- |
|  |  | Area (km^2^) | % Area | Area (km^2^) | % Area | Area (km^2^) | % Area | Area (km^2^) | % Area |
| Chimpanzee | Unsuitable (0-0.2) | 60.5 | 2.9 | 175.6 | 33.3 | 781 | 63.2 | 749.6 | 90.5 |
|  | Low  (0.2-0.4) | 716.5 | 34 | 272.5 | 51.7 | 390.4 | 31.6 | 62.5 | 7.5 |
|  | Moderate (0.4-0.6) | 1083.5 | 51.4 | 64.6 | 12.3 | 58.3 | 4.7 | 7.9 | 0.9 |
|  | High  (> 0.6) | 246.2 | 11.7 | 14.2 | 2.7 | 5.4 | 0.4 | 8.6 | 1 |
| Gorilla | Unsuitable (0-0.2) | 3.4 | 0.2 | 27.6 | 5.2 | 316 | 25.6 | 167.9 | 20.3 |
|  | Low  (0.2-0.4) | 361.8 | 17.2 | 128.8 | 24.4 | 423.1 | 34.3 | 291.9 | 35.2 |
|  | Moderate  (0.4-0.6) | 1458.5 | 69.2 | 257.6 | 48.9 | 407.9 | 33 | 340.1 | 41.1 |
|  | High  (> 0.6) | 283.1 | 13.4 | 112.9 | 21.4 | 88 | 7.1 | 28.6 | 3.5 |

**Table S2 cont**'**d.**

| Species | Class | ZIC 28 or UFA 10–007 | | ZIC 29 or UFA 10–009 | | ZIC 30 or UFA 10–012 | | ZIC 31 or UFA 10–064 | |
| --- | --- | --- | --- | --- | --- | --- | --- | --- | --- |
|  |  | Area (km^2^) | % Area | Area (km^2^) | % Area | Area (km^2^) | % Area | Area (km^2^) | % Area |
| Chimpanzee | Unsuitable (0-0.2) | 106.4 | 13 | 401.8 | 23.2 | 44.2 | 6.1 | 897.1 | 81.6 |
|  | Low  (0.2-0.4) | 545.8 | 66.8 | 1132.5 | 65.4 | 654.4 | 90.4 | 187.7 | 17.1 |
|  | Moderate (0.4-0.6) | 120.6 | 14.8 | 195.9 | 11.3 | 24.9 | 3.4 | 14.6 | 1.3 |
|  | High  (> 0.6) | 44.1 | 5.4 | 1 | 0.1 | 0.4 | 0.1 | 0 | 0 |
| Gorilla | Unsuitable (0-0.2) | 2.1 | 0.3 | 106 | 6.1 | 0 | 0 | 27.1 | 2.5 |
|  | Low  (0.2-0.4) | 235.9 | 28.9 | 314.6 | 18.2 | 43.3 | 6 | 383.2 | 34.9 |
|  | Moderate  (0.4-0.6) | 469.4 | 57.5 | 1201.7 | 69.4 | 622.7 | 86 | 661.8 | 60.2 |
|  | High  (> 0.6) | 109.5 | 13.4 | 109 | 6.3 | 57.8 | 8 | 27.3 | 2.5 |

*Total proportion of high, moderate, low and unsuitable chimpanzee habitats within FMUs = 1.4%, 7%, 47.2% and 44% respectively. Total proportion of high, moderate, low and unsuitable gorilla habitats within FMUs = 8.9%, 56.6%, 25% and 8.6% respectively.

*These values were obtained by calculating the average proportions of each suitability within the 7 FMUs.

(a)

(b)

**Figures S8.** Comparison in chimpanzee and gorilla habitat suitability between the Lobéké National Park and its surrounding FMUs; (b) Chimpanzees; (c) Gorillas
